# ‘Do-It-Yourself’ Healthcare? Quality of Health and Healthcare Through Wearable Sensors

**DOI:** 10.1007/s11948-016-9771-4

**Published:** 2016-03-30

**Authors:** Lucia Vesnic-Alujevic, Melina Breitegger, Ângela Guimarães Pereira

**Affiliations:** Joint Research Centre, European Commission, Via E. Fermi 2749, 21027 Ispra, Italy

**Keywords:** Knowledge assessment, Self-tracking, Wearable sensors, Digital health

## Abstract

Wearable sensors are an integral part of the new telemedicine concept supporting the idea that Information Technologies will improve the quality and efficiency of healthcare. The use of sensors in diagnosis, treatment and monitoring of patients not only potentially changes medical practice but also one’s relationship with one’s body and mind, as well as the role and responsibilities of patients and healthcare professionals. In this paper, we focus on knowledge assessment of the online communities of *Fitbit* (a commercial wearable device) and the *Quantified Self* movement. Through their online *forums*, we investigate how users’ knowledge claims, shared experiences and imaginations about wearable sensors interrogate or confirm the narratives through which they are introduced to the publics. Citizen initiatives like the *Quantified Self* movement claim the right to ‘own’ the sensor generated data. But how these data can be used through traditional healthcare systems is an open question. More importantly, wearable sensors trigger a social function that is transformative of the current idea of care and healthcare, focused on sharing, socialising and collectively reflecting about individual problems. Whether this is aligned with current policy making about healthcare, whose central narrative is focused on efficiency and productivity, is to be seen.

## Wearable Sensors at Crossroads: The Technology and Healthcare Policies

### Digital Health Technologies and European Policies

Medical sensors have a long history of usage in medicine and healthcare (Wilson 1999; Aberg et al. 2002; Ko et al. 2010). These portable devices collect specific data from the body, for example blood glucose levels, heart rate or movement. They are used for fitness and/or health purposes and are often advertised as devices that will motivate people to exercise more, help losing weight and so on. In parallel, various studies (Pantelopoulos and Bourbakis 2010) have been conducted for assessing the usefulness of these devices in healthcare, by providing real-time information about a person’s health while the patient and medical doctor are not at the same place. Available as small wearable sensors, wearable material, smart textile or implantable sensors, these devices monitor heart rate, blood pressure, body temperature, respiration rate, etc., working through a user interface to algorithms through which personal data is extracted and processed (Pantelopoulos and Bourbakis, *Op. cit.*).

During the past decade, wearable sensors have become very popular, due to rising healthcare costs and an ageing society that has become a serious problem (Shah 2009). Based on the analysis of fairly recent European policy proposals in the domain of telemedicine and healthcare (COM, European Commission 2014), it seems as though the new technology appeared as deus ex machina for policy makers who see in it a pathway to lower the costs of traditional healthcare and improve the quality of care delivery. Wearable sensors are often seen through the imaginary of preventive care where these devices could help people to transform their unhealthy lives. Ayo (2012) argues that the number of health policies that focus on healthy lifestyles promotion throughout the world has considerably increased in the last 30 years, as a consequence of neoliberalism and the so-called ‘healthism’^1^ a new lifestyle where individuals become obsessed with their own health and consciously work on it. Thus, healthism contributes to the vision that a responsible citizen should adopt lifestyles described as healthy (Ayo 2012:100). The rise of digital technologies in the domain of healthcare has been critically studied from social, cultural, political and ethical vantage points by various scholars (e.g. Ayo 2012; Lupton 2014; Wright and Halse 2014). For example, the Internet facilitates the access to health information for all citizens, with the Pew Research Centre stating that 8 in 10 Americans look for their health information on the Internet (Lupton 2014). These and similar practices ‘persuade individuals to monitor themselves and others by increasing their knowledge of food and health and by instructing them on how to change their lives by eating healthily and staying active’ (Wright and Halse 2014, p. 839).

Lupton (2014) argues that new technologies are, in general, ‘intensely political’ (p. 2) changing power relations as they influence social relations and institutions, allowing new digital inequalities and new spaces for surveillance to arise. Surveillance is understood here as monitoring by digital devices and sensors. For example, from this perspective wearable sensors for healthcare fit well with what the influential French philosopher Gilles Deleuze described as ‘societies of control’ (Deleuze 1990). In ‘societies of control’ power lies with technologies, which multiply the possibilities for controlling our freedom (Deleuze, *Op. cit.*); the Foucauldian model of the ‘panopticon’ (1975) no longer describes the surveillance mechanisms where our online activities are monitored by distributed corporate and institutional agents in the form of algorithms that *live* in the devices we use for communicating, searching, self-tracking and monitoring. The ubiquity and connectivity of the devices are being organised through what is described as the Internet of Things (IoT),^2^ a searchable network of physical and virtual ‘things’, in which the subjects and objects of telemedicine or mobile health are just a part. We see the IoT as a large project of surveillance, where Deleuze’s ‘dividuals’ are the fragmented pieces of data that are processed invisibly and unnoticeably resulting in the impairment of human agency. In the neo-liberal tradition self-tracking can be seen as self-governing, becoming ‘a strategy for producing responsible citizens who take care of their own health consistent with state objectives’ (Till 2013). Existing and emerging devices and apps provide new ways of self-monitoring, *self*-*veilling* (Boucher et al. 2014) and voluntary or involuntary sharing our data with others in the network. But as Lupton (2014) argues, users participate in a corporatised context where the developers decide how data are generated, manipulated and used and are able to prevent users from accessing their own data (p. 7). Although we agree with the potential for control and surveillance through self-tracking apps and devices, we argue that these sites also offer new possibilities for resistance and empowerment, especially when users are producing their own methods and means for self-tracking, as explained by Anne Wright in her ‘Body Track’ project.^3^ Thus, we focus on knowledge production through *self*-*veillance* and self-care and how these practices relate to traditional healthcare and linked policy proposals.

### Self-Tracking: Issues of Quality

Self-tracking refers to the systematic recording and analysing of information about one’s own health, diet or different activities, by using technology in obtaining the data. It is mostly connected to the use of wearable sensors that serve for data gathering.

In a lecture about self-tracking,^4^ Anne Wright, the leader of the Body Track project, argued that self-tracking is useful because medical solutions that fit the majority do not fit everyone. According to Wright, self-tracking is also helpful for finding the cause of a certain condition that a diagnosis does not reveal: ‘Taking an action based on our own experience can be more powerful than doing it on your doctor’s recommendation’.

In other words, self-tracking helps us understand what is going on with our body and how to make better choices. The main steps followed by users are observe-hypothesise-act-reflect-adjust-iterate. The main justification (Remen 2006) for digitally recording these types of data is that patterns are hard to recollect from memory, and in that sense self-trackers construct a personal narrative. Wearable sensors start as an extension of human senses but ultimately they seem to be designed to substitute for them.

In this paper we look at wearable sensors user communities’ discussions in order to assess the quality of knowledge created and shared about wearable sensors in connection to health and healthcare issues. Through the online forums we investigate how user’s knowledge claims, shared experiences and imaginations about wearable sensors interrogate or confirm the narratives through which they are introduced to the publics. We also explore the connections between forum users and traditional healthcare systems as well as between users and policy making in the domain of healthcare.

With this perspective we examine the wearable sensor Fitbit and Quantified Self movement forums. Through both forums, users of wearable self-tracking devices discuss diverse issues, including sensor related technicalities, meanings of ‘health’ and ‘healthy’, developing into a community around those topics. Whilst we are not comparing these two forums, we would like to use them as two cases exemplifying the phenomenon of self-tracking discussed here. Therefore we have used the same approach to both.

Fitbit is a small wearable device produced by a US-based company with a global reach, founded in 2007, with the mission of empowering and inspiring people ‘to live a healthier, more active life’ as expressed in their mission statement. Fitbit records the number of daily steps walked and floors climbed. The general purpose of Fitbit is to assist and encourage users to increase their physical activity in order to improve health. Additional features are sleep monitoring (the tracking of movement during sleep) and the possibility of comparing calories consumed and calories burned. The Fitbit user is asked to wear the device 24/7, insert food consumption and activities other than walking, as well mood swings in a cloud-based platform. As a result the user can see charts with information on daily activity, resting, eating and compare the data over a time span and see how ‘fit’ he or she is compared to other Fitbit users. This is an example of what Foucault refers to as the technologies and practices of the self, where the agency of the subject to influence their own bodies, thoughts etc. is accentuated “as to transform themselves in order to attain a certain state of happiness, purity, wisdom, perfection, or immortality” (Foucault 1988:18).

The Quantified Self is a US company that supports a world-wide community of users and makers of self-tracking tools which describes itself as a movement. In an interview we made in 2013, Adriana Lukas, the organiser of QS meetings in London, says: “There are no institutions or businesses involved but just individuals doing things because they are trying to solve a problem, and that is one of the most important points about Quantified Self that I can give you.” Although it is not completely clear how the ‘movement’ and the company link together, it is interesting to note however, that the coordinator of the UK movement does wish to indicate that this is an initiative that is grounded in actual needs of people and that there are no institutions involved.

### Knowledge Assessment: Quality of Knowledge Through Wearable Sensors

We will introduce here two concepts we use in our research, ‘knowledge assessment’ as a means to critically examine knowledge produced in loci other than the scientific realm and ‘extended peer review’ the process by which relevant knowledge applied to address a particular issue is scrutinised. Knowledge assessment (see Funtowicz 2006) activities aim at assessing the quality of the processes of knowledge creation and its products deployed to underpin action. It must be noted that ‘knowledge’ here does not map onto ‘scientific knowledge’, but includes types of knowledge created in different spheres of life and experience. In this context, the existing quality control in research science cannot be applied. The evaluation of quality of knowledge in terms of fitness for purpose (quality being a relational attribute) is the core of knowledge assessment activities. But who determines what is fit for purpose? The concept of ‘extended peer community’ as articulated by Funtowicz and Ravetz in their descriptions of post-normal science^5^ is relevant here. The concept emerges from the recognition that for current types of policy-relevant science, the maintenance of scientific quality depends on open dialogue between all those affected. Today, encouraged by the online opportunities, ‘those affected’ combine their current knowledge with creative and experienced ‘extended facts’ (Funtowicz and Ravetz, *Op. cit.*) in order to actively scrutinise and influence what is being proposed to them. This can be seen in a variety of endeavours, where an increasing number of issues are raised outside the traditionally accepted legitimate and credible knowledge spheres, be that about a health policy issue (see for example the Cochrane collaboration) or crowdfunding activities to develop or implement a ‘do-it-yourself’ project (see e.g. kickstarter or goteo).

Here, we extend knowledge assessment activities to processes of technology development and deployment in which meanings and issues do not emerge through a formal policy cycle but rather arise in organised forums of discussion among user communities (the ‘extended community’) of technological artefacts—such as wearable sensors technology. The categories for assessing the quality of the debates within user communities will focus on knowledge produced through experiences, opinions, skill development, etc., documented by user’s interactions. Here, we will borrow the ‘pedigree’ of the quantitative information concept first developed by Funtowicz and Ravetz (1990) in their NUSAP system^6^ and then later extended by Corral Quintana (2000) to qualitative information. Pedigree “is an evaluative description of the mode of production (and where relevant, of anticipated use) of the information” (Funtowicz and Ravetz, *ib idem).* We interrogate quality using questions such as: who is making statements? Where does the information come from? Who is listening? What legitimacy or credibility mechanisms are sought? Etc.

## Chasing the Pedigree…

In order to apply knowledge assessment methodologies we examine online Fitbit users communities’ discourses focusing on the quality of knowledge produced and shared about wearable sensors in that context. The online users’ community forum has a large number of entries around many topics, grouped by theme. At the same time and separately from Fitbit, the *Quantified Self* movement has a similar online forum where self-trackers gather as an online community discussing various topics in a similar way to Fitbit users. Conducting our research in both forums allowed us to dissociate from a particular gadget and focus more on the self-tracking aspects, as the QS movement is not associated with any particular brand. As we examined the users’ posts in order to capture elements for knowledge quality assessment, a progressive focus was used to choose themes that could best exemplify the knowledge assessment application.

As introduced earlier, in our study we focused on the analysis of pedigree of qualitative information. A number of quality categories were seen as appropriate for the type of material available from the user community forum. Having the quality categories in mind we looked for framings, factual or imagined argumentation, justifications, motivations, suggestions, appeals, assumptions and other narrative elements in the stories shared in those posts.

Two main quality categories were found to be appropriate for evaluating information pedigree in community forum: fitness for purpose and reliability. Table 1 summarises categories and subcategories.

**Table 1:** Analysed categories and subcategories

Category	Subcategory	Observations
Fitness for purpose	Relevance: correspondence between information used and issues addressed Accuracy of information Comprehensiveness of information	Here we look at strategies by users to ensure information provided fits intended objectives of discussions started
Reliability	Sources of information to support knowledge claims Sources of legitimacy	Here we look at strategies used by participants to legitimise information ‘offered’

We looked at these forums from the perspective of digital ethnography (Domínguez et al. 2007), by observing and examining participants’ discussions in these places. The most important advantages of the unobtrusive observation via the Internet and other traditional methods such as interviews, focus groups or experimental research are the richness of the collected data and the frankness of participants which is more difficult to obtain in face-to-face conversations (Hine 2011). We have used explorative quantitative and qualitative analysis of the content posted first on the Fitbit forum and later on the Quantified Self forum. This analysis is followed by in-depth qualitative analysis with knowledge assessment methodologies described earlier.

### Mapping the Topics

In this section we present the most commented topics on the forums we have analysed, grouped into several categories for further analysis.

#### Fitbit Forum

Fitbit offers its users access to a user community forum where people can exchange their experiences and ask for advice from other users. The forum is structured in six groups: Announcements, Big Losers, Food suggestions, Feature suggestions, General, and Help and Support. The ‘Big Losers’ category is for discussions of users that are trying to lose or have lost 75 pounds/34 kilos or more. In the ‘Food suggestions’ and ‘Feature suggestions’ categories, users are asked to provide input for the food database of Fitbit and for the development of features of the Fitbit device. By mapping the topics discussed in all the different groups, taking into account all the topics that were commented on a time period of 10 weeks (30.4.2013 to 9.7.2013), we could identify 15 categories of topics. In total 374 topics were posted in the named time period (Table 2).

**Table 2:** Fitbit forum

Category	Number of topics	Topic examples
Building communities/finding friends	137	‘Hello from Seattle WA!’, ‘Group Wanted/Woman, Late 40s/100 to lose’, ‘Guys talking about Guy Stuff and Getting Fit’
Asking for advice from the community	78	‘Need a person to teach me what I’m doing wrong to get this weight off’, ‘What to eat before work out’, ‘Frustrated and want some advice’
Sharing personal experience	36	‘I have lost over 100 lbs!’, ‘Just hit my first goal’
Technical features	34	‘Please enable Bluetooth syncing with Windows Phone 8′
Suggestions for improvement of Fitbit	28	‘Would be great to add a Recipe feature’
Asking for information	17	‘How many steps in a mile?’
Recipes	9	‘Share your Recipes!’
Health	6	‘Fitbit heart rate monitor’
Other (offering help, posts by Fitbit, feedback for Fitbit, recommendations, trading wristbands, goals, stupid things)	29	

Looking at the number of topics in each category, we can see that the main concern of Fitbit users who post in the forum is to find friends and build communities. They seem to be looking for other Fitbit users in a nearby geographical location (‘Hello from Seattle WA!’), users with the same goals about weight loss and other common issues (for example ‘Group Wanted/Woman, Late 40s/100 to lose’ or ‘Guys talking about Guy Stuff and Getting Fit’) or just search friends in general who can support them by losing weight together. Another large group of topics is centred on advice on weight loss. The Fitbit users ask their fellow users’ advice on dieting, exercise and how to stay motivated (‘Need a person to teach me what I’m doing wrong to get this weight off’, ‘What to eat before work out’, ‘Frustrated and want some advice’). Many people also use the community forum to communicate their personal experience, without directly asking for advice or support. Mostly these posts are related to successful weight loss or increased fitness (for example: ‘I have lost over 100 lbs!’, ‘Just hit my first goal’). The suggestions for improvement of Fitbit are mostly focused on design, the development of the food database (where users can log their food consumption in order to calculate the daily calorie intake), and technical features. The latter concerns, for example the possibility to synchronise Fitbit with smart phones and other tracking devices and to include additional features like bar code scanning to make food monitoring easier.

#### Quantified Self forum

The Quantified Self forum is structured in 16 topics, with the greatest number of posts in the following threads: Apps & Tools, QS Open Forum, Sleep, Learning and Cognition. We mapped the topics discussed in different threads by taking into account all the topics commented on the period of 10 weeks (27.1.2014 to 6.4.2014). We have identified 10 categories of topics. In total 124 topics were posted in the named time period (Table 3).

**Table 3:** Quantified Self forum

Category	Number of topics	Topic examples
Building communities/finding friends	8	Hi everyone, Hello from Grand Bay
Posts by QS	3	A new tool guide for the QS Community?
Sharing experience	14	Mood tracking methods?, KYou and Konnectors: enable the Personal Data Task Force
Asking for advice from the community	45	Zeo Sleep; Food Database; Tracking Pain/Discomfort-Thoughts?; Dealing with people privacy
Recommendations	5	Learning Tracker; Sleep Cycle
Advertising own project	18	Tallyman; Nike Sense
Call to participate in research/event etc.	24	Test out a new app for sleep improvement; Wear a pedometer or track your fitness? Help UW Researchers!
Sharing information	4	Sun sensor
Buy/sell	1	Seeking 10 Fitbit Flexes
Experiments	2	Nootropics?; QS Experiment

As we can see from Table 3, the majority of posts are connected to asking for advice and sharing experiences with others. There are also a considerable number of calls to participate in research, launched by independent research institutions or companies, but this remains out of our scope. The QS forum is used less than the Fitbit to find friends, but more to exchange ideas on specific topics. Instead of losing weight together, the main issue is how to use or further develop self-tracking tools, the self-experiments, sharing experience with others and asking for advice on how to proceed with the experiment/self-tracking that they are conducting. Instead of adding friends in a social network as in the Fitbit community, the QS movement organises *official* meetings in locations where there are enough people involved in the movement. An interesting feature of QS forums is not the number of posts, but rather the large number of associated views and comments. For instance, although the ‘Zeo shutting down: export your data’-thread contains 305 replies, it has at the same time 59,027 views. Similarly the thread ‘Try my latest sleep hack’ has only one comment but 13,336 views.

### Quality in the Quantified ‘Self’

In this section we provide the main types of quality issues that arise from the analysis of both the Fitbit and QS forums.

#### Fitbit

In our analysis we have focused on forum entries and threads that included some form of suggestion for the improvement of the wearable sensor technology, both in functional and usage terms, as well for the improvement of the knowledge base associated with the use of Fitbit and similar technologies. We exemplify below the types of thread contribution strategies used by participants in the threads we chose to analyse in more depth: ‘health at any size’, ‘non-wireless option’, ‘smoking log’, ‘1,750 C/day deficit…’.

In some threads forum members make connections between Fitbit technology and wider issues of health, thus initiating wider discussions about the place of wearable sensors in healthy lifestyle and health in general. Through this activity, users are interrogating different meanings of this technology. In the examined threads, users extend and connect the thread topics through posts with titles such as “a health program developed by a doctor”, “the creator of HAES [a health program] did a head-to-head study” and so on. And in a response to these posts, a wider connection to health is further discussed, as in the following post:

I had never heard of HAES until this post, but based on your explanation in a previous post, I definitely support that philosophy in my life. (…) I have more energy and feel better […] since I have started logging my food (early July), I have a much healthier relationship with food.—*‘Health at any size’ thread of Fitbit community*—*response to User 1HAES.*

As the previous example shows, in this post the user connects the use of logging food consumption and the measuring of weight as an indicator of health. Importantly, these connections are not documented with references to scientific or other authoritative material, but only based on personal experience.

In many entries to the richer debates, we can see competent observations and motivated suggestions, sometimes using specialised knowledge, derived from the participants’ professional walks of life, who intervene with mixed ‘hats’, sometimes attempting to legitimise their entries through the professional hat—replying as a pharmacist, for example. The quotes below illustrate this observation where users make suggestions by reasoning in terms of health, legal, and practical terms, as the performance of authority is enacted:

I’d like to see a wireless-free option. Why? My job often takes me into places where no wireless devices are allowed. Sometimes it’s enough to be able to turn off wireless, but in most, wireless devices are completely forbidden.—*Non*-*Wireless thread of Fitbit community* — *User 1NW.*

There are many such quotes where the user attempts to legitimise the opinion through professional credentials. They present themselves as medical doctors, Ph.D. students, friends of medical school students, pharmacists and so on. Also, they try to discredit people who use a device (or a measurement) the “wrong way” by stating that they are “laymen”.

On the other hand, some observations in need further justification or documentation, exhibit only the ‘I like’ corroboration. For example, there are strong claims without any specific documentation or attribution to any legitimate source. They often start with “all scientific studies…”, “physics provides a guarantee…”, “there is virtually no chance whatsoever…”, “most guidelines say…”.

In fact, many assertions correspond to auto-ethnographies including experienced facts or personal or common popular views. For example, on the “smoking log” thread a suggestion for functionality that monitors quitting smoking is made and many forum users have responded with personal experience to what they see as an improvement: “I, like many others have joined the Fitbit community in an effort to improve my overall health”, “seeing and tracking this activity should help make us confront the unhealthy habit”, “what I found the most helpful “, “I think that the standard basal metabolic rate formula just doesn’t seem to work for me. I guess I have experimental evidence (…)When I started logging my food on this site I noticed…”

In some cases further information or references are given, including rectifying information given through referencing or adding further information such as: “She also ran one of the really important clinical trials in the field…” or “Read Why We Get Fat and What We Can to About it by Gary Taubes…”.

In other cases those who follow the thread ask for evidence in the form of further information or references, after having expressed doubt about the plausibility of the assertions. Hence, the strategies for quality assurance of what is discussed and offered sometimes follow the traditional schemes of quality control in science. For example, regarding the “health at any size” thread, a user requests specifically peer-reviewed publications, considering other sources irrelevant and illegitimate:

I’m also sceptical of the above claims about the HAES vs. other diets study. It would be helpful if someone could point me to a (peer-reviewed) publication on the study and not a media report.—*‘Health at any size’ thread of Fitbit community* — *response to User 1HAES, introducing himself as a PhD student.*

#### Quantified Self

The QS forum is somewhat different to the Fitbit forum. The thread with the most comments is ‘Apps & Tools’. It is used by QS members, but also by developers that want to get feedback on new apps. Some of the QS forum members are not only users but also participate in creating/designing apps or devices that can help self-tracking (as well as the platforms, as we have seen in the Body Track project). This is an interesting difference from the Fitbit forum, as users turn into producers, while incorporating their own personal experience in the creation of an app. Despite the unpaid work in which users are enrolled, the inclusion of users in testing and contributing to the production of devices can be seen as very positive because they lead to a greater public engagement. These posts usually start in a similar way to this one: “A […] neuroscientist with a focus on sleep approached me to help him make an app […] I’d be really happy to get some feedback from QSers.”

Or, the app creators post a direct message to users such as:

We are going to conduct a formal study with it — so let me know also if you’d be interested in testing it out […]’*Test out a new app for sleep improvement thread of QS community User dgartenberg.*

Some entries ask for further information, reference or clarification to what was previously posted. The forum members want to assess the knowledge that appears in other members’ quotes by looking for themselves at the background information and by searching for the pedigree of information:

Do you have more background information that you can post here? Portland’s QS group is planning to do a meet up focused on sleep, might be worth checking out? *Test out a new app for sleep improvement thread of QS community* —*response to User EJain.*

In some cases further references are given. Sometimes they rely on self-testing or they refer to relevant medical literature or studies conducted in the same field. Or they just give examples such as: “More information can be found at the website:[…]”; “Here is info on the science behind it…”; “These findings were published in…”.

Discussions where people use auto-ethnography are also very common. By talking about their health problems and self-experimentations, users try to solve the medical problems they have by different (un) verified methods and often ask for the help of others based on their experience and consequently share their own problems and experiences. After listening to the experience of others, they make a conclusion about their own conditions:

Every morning, I wake up with one nostril almost completely congested, and the other free. Is this normal? (…) I went to four doctors about ….Of course, this is highly dubious…, I would like to ask for help from my fellow quantified selfers…—*Poll: how often do you wake up with a nostril congested? Thread of QS community User Mike.*

After having received answers from different users, the user who initiated the thread concludes that he has a rare condition and therefore medical doctors cannot help him. He sees a solution in self-experimentation followed by quantification.

‘Thanks everyone for their replies[…] It seems like I have a rare-ish condition.…. I’ll have to quantify how that works. …’*Poll: how often do you wake up with a nostril congested? Thread of QS community* —*response to User Mike.*

There are also many examples of auto-ethnographic studies that people conduct in order to prove/disprove an accepted *truth* or someone’s assumption. This quote illustrates the emerging idea of Do-It-Yourself medicine, where the accent is on our own body and a personalised approach with the goal of finding a proper cure based on experiments that is hoped to show what works the best.

Final result: it negatively affects my sleep, *d* = – 1.1. For comparison, most improvements score less than 0.5. See [link]. If I were to do a follow-up experiment, it would be blinded & randomised as usual, with consistent doses (eliminating objections 1–3), but more importantly, the dose would be consumed upon awakening. It is highly unlikely I will bother with a follow-up experiment…- *Potassium citrate hurts sleep?Thread on QS forum* — *User gwern*.

## Discussion

In this paper we have used knowledge assessment methodologies and in particular pedigree analysis, to perform a quality check of knowledge produced by user communities of wearable sensors. We have looked into two different forums that respond to the ideas of self-care and DIY health; one forum associated to a well-known branded sensor and another forum of a self-organised community that is not linked to a particular wearable sensor brand.

This approach allowed us to assess the quality of information posted online where users discuss their own experiences, experimentations, make suggestions to others, etc. We have considered two main categories to look at the threads and individual posts: fitness for purpose and reliability of information exchanged, including the strategies through which individuals seek to communicate their learning, to make credible their knowledge claims, to justify their assumptions and to make plausible their own heuristics. Users are here seen as part of the ‘extended peer community’ that actively debates, examines and decides upon scientific or technological issues, health or regulatory related, that have been proposed to them by corporations and public institutions. Table 4 illustrates our findings, summarising the results obtained through the analysis of both forums showing the main types of quality issues that arose.

**Table 4:** Summary of quality issues arising from the analysis of the two forums

Category	
*Fitness for purpose*	
Relevance	*Fitbit and QS:* all posts that we have analysed are relevant for the issue raised in the first place
Accuracy	*Fitbit and QS:* different levels of accuracy are encountered, but in general posts are not well documented and are based on users’ own experience; no verification is possible
Comprehensiveness	*Fitbit:* many of the entries are not well documented; in some cases important claims are made but they are not developed neither supported by traditional science nor by ‘extended facts’ *QS:* many entries are documented with self-experimentation
*Reliability*	
Sources of information	*Fitbit and QS:* typical sources are: life logging and their own experience webpages of organisations or commercial books media pieces In many cases references are asked for; other times many claims go unverified but obviously it is not clear what the users do with the information The majority of QS sources are life loggings and their own experience
Sources of legitimacy	*Fitbit and QS*: the majority of users do not use any form of authoritative strategy apart from their own lived experienced to reply in the threads Some users introduce themselves as ‘experts’ to bring legitimacy into their replies Some users refer to external references, that being people or peer-reviewed materials

The content of the entries in the threads analysed are quite competent and discussed, often based on personal experience and auto-ethnography. Often however, the context in which the experience is told is not described or accounted for. Hence, albeit these exchanges may fit the purpose of maintaining a dialogue where other users can intervene, add, rectify or process otherwise, they might not entirely respond to the issue launched in the thread. The posts are mainly auto-ethnographic and are mostly relevant for other participants of the forum.

The types of legitimacy sought by users varies and includes other sources of information, allusion to well-known public figures and work, not necessarily peer-reviewed scientific sources. We would argue that the value of these threads is precisely the experiential facts brought into the dialogues and the issues raised around the thread’s theme: the particular wearable sensor technology and ultimately the overall use of wearable health technology. We found that the quality control, and specifically the pedigree analysis of the entries, was not more difficult to analyse than many official ‘expert’ documents suffering from similar weaknesses. For example, a common snag of the European Union papers is self-referencing, i.e. the European Commission (EC) justifies certain types of claims that would require references to expert studies, with arguments that were made in earlier EC publications of a policy nature instead of referencing external expert sources of information (Breitegger et al. 2014).

Knowledge assessment also allowed us to examine the motivations of citizens to engage with self-tracking, self-care and health *self*-*veillance* through wearable sensors. For many citizens, monitoring their own health, through different devices or apps available in the market is a response to actual needs, health related but also because these devices respond to other needs when it comes to health and wellness, such as that of conviviality. These devices belong to a generation of devices that are packed with lifestyle narratives implying strong normativities and imaginaries in the form of tempting proposals for how one should live one’s life. But, in an interview with the authors, the London coordinator of the Quantified Self movement in London, stated:

‘The people that I work with are constantly pushing the boundaries of what can be done and how they experiment and they do not care about data per se but about solving their problems. Let us say that you have diabetes or you are interested in running an ultra marathon or you have Parkinson’s Disease and you need to measure certain markers, you want that. You want to make your life better and do not really care about fancy marketing, such as with Fitbit, which is banking on lifestyle marketing and wellbeing and so on.’

Furthermore, it is worth noting that users seem to be engaged in an experiment with multiple purposes: whilst they actually and objectively contribute to the fine tuning of the technology, they also are engaging with the deeper experiment of non-medical devices being used to perform a health function giving the user a sense of agency with regard to their health. The added agency is provided by, on the one hand the use of the personal(ised) device and on the other hand the emphasis placed on sharing and on the idea that self-tracking is a ‘collective endeavour’ to address common health issues. Therefore wearable sensors serve purposes other than just self-monitoring and DIY health, including social purposes, such as sharing, mutual learning, going deeper in issues and companionship. This is transformative of the prevalent narrative of healthcare, which seeks for efficient, cost-saving, technology driven, virtual connectivity and patient-centred approaches away from clinical settings.

In this way the wearable experience becomes actually the testing out in vivo of health and wellbeing narratives at a broader level.

‘There is no self in the quantified self, and that is not because it cannot be measured. It is because you cannot get the data out of the varied sensors, applications and platforms and combine them into something that approximates [the] self more than just one data stream. (…) An aspect of self measurement is a combination of all those data streams and at the moment you cannot measure that.’*London Quantified Self coordinator in an interview in 2013.*

## Conclusion

The self-tracking gadgets attract different audiences, including those trying to lose weight, athletes, hypochondriacs and so on (Waltz 2012). Hence, by sharing their experience, citizens may be focusing on resolving their own problem and not necessarily buying into a lifestyle and consumption narrative sold with the devices they use.

Another important narrative with which these devices are proposed to citizens is empowerment; this is also visible through many of the discourses in the threads as we have seen and aligns well with policy discourses in healthcare in the European Union (see for example the mHealth green paper (European Commission 2014)).

In what seems to be a transition to an era of do-it-yourself healthcare, citizens seem to be implicitly and explicitly asked to rely more and more on their own observations, searching for information online instead of consulting a doctor. Given the price of healthcare which has been growing considerably, this sort of patient-driven healthcare model is already finding its place in policies on healthcare worldwide (Wolbring and Lashewicz 2014). Wearable sensors, social media, ubiquitous computing, VoIP (Voice over Internet Protocol) are now seen as leading technologies in home health monitoring and care (see e.g. Wolbring and Lashewicz, *Op. cit.;* Afshar 2014).

In fact, there seems to be a co-produced need and agreement for a ‘do-it-yourself’ strategy to tackle one’s health; but what the analysis of these threads show is that the ‘community’ forming around the discussion of issues related to wearable sensors is as important as the self-monitoring. Whilst self-tracking, i.e. reducing to a small number of parameters our health and fitness, is seen as an improvement of quality in care, the ideas of socialising and sharing experience about health issues change fundamentally the experience of ‘do-it-yourself’ care and knowledge production around health issues.

This interesting remark arising from a phase of knowledge assessment that we have reported elsewhere (Breitegger et al. 2014) tells us much about the expectations of users from technology and from measurement. Reducing the ‘self’ to a compounded set of data sets seems to be in the greatest imaginary of all: for users but also for business and policy making. In contrast, it is interesting to note that those reductionisms entertain deep conversations about health and healthcare, as we have seen from the sample quotes from the forums we have analysed.

But there are more alignments that emerge from the pedigree analysis that we have conducted; for example, as explained in the introduction, policy makers and other stakeholders argue that there is a need for reducing healthcare costs. According to the efficiency narrative that supports healthcare in the EU, this is perhaps achievable through greater engagement of citizens in self-care practices. There seems to be no evidence, however, about how knowledge produced in realms such as citizen online forums actually reaches or is heard by policy and business actors. Yet, what needs to be understood is that healthcare is no longer entirely in the hands of institutions that practice, regulate or provide healthcare but also in the hands of citizens who resolve their problems in active ways, acting sometimes as guarantors of quality of the healthcare system. In fact, citizen initiatives like the QS movement claim the right to ‘own’ the sensor generated data. But how these data are fit for purpose in traditional healthcare systems is an open question—see, for example, Ko et al. (2010); Redmond et al. (2014).

Funtowicz (2006) argues that for the policy-relevant scientific problems, there is a need for an ‘extended peer community’ to be included in the process of decision and policy making, through an open dialogue that would include diverse stakeholders including citizens. Would this be a commitment for enhancing quality of policy making? We would rather conclude that current disconnects on practice, expectations and actual appropriation of these technologies show that the main postulates of post-normal science, quality assurance through deliberation among different partners in the process, still needs to be accepted by not only researchers and citizens but also policy makers and the industry.

## Footnotes

1 A term created by political economist Crawford (1980) to describe the political ideology promoted in the USA in the 1970s where health is transferred to the level of individuals. Skrabanek (1994) uses it to describe the government’s promotion of, and attempt to establish, a healthy lifestyle. Ayo (2012) describes *healthism* as a new lifestyle where individuals become obsessed with their own health and consciously work on its improvement through the combination of wellness and fitness programs (such as, e.g. Jane Fonda’s workout videos in the 1980s).

2 The Internet of Things (IoT) (or *Internet of Everything* as described by CISCO Corp.) refers to the fusion of the “real” physical and virtual worlds. Guimarães Pereira et al. (2013) define it as “as a global network infrastructure, linking uniquely identified physical and virtual objects, things and devices through the exploitation of data capture (sensing), communication and actuation capabilities” (p. 8).

3 The Body Track project is a self-tracking open source project that will be available to everyone for free.

4 Wright, Anne (2014). ‘Self-tracking. Reflections from the Body Track project’. Lecture at European Commission Joint Research Centre Ispra. 24 March 2014.

5 Post-normal science is a concept, attempting to characterise a methodology of inquiry that is appropriate for cases where ‘facts are uncertain, values in dispute, stakes high and decisions urgent’; it was first described in relation to environmental issues but its application has since been extended to other areas of policy.

6 NUSAP is a notational system proposed by Funtowicz and Ravetz (1990), which provides an analysis and diagnosis of uncertainty in science for policy. It captures both quantitative and qualitative dimensions of uncertainty and enables one to display these in a standardised and self-explanatory way. It promotes criticism by clients and users of all sorts, expert and lay and will thereby support extended peer-review processes.
